# Supplementary material for: Solar-driven selective conversion of millimolar dissolved carbon to fuels with molecular flux generation
Source: Nat Commun. 2025 Feb 12;16:1558. doi: 10.1038/s41467-025-56106-3 (PMC11821833; doi:10.1038/s41467-025-56106-3)
Supplement: Supplementary file 1 — Supplementary Information [file 41467_2025_56106_MOESM1_ESM.pdf]

**Supplementary Information**

# Solar-driven selective conversion of millimolar dissolved carbon to fuels with molecular flux generation

Bin Liu[1, 2], Zheng Qian[1, 2], Xiang Shi[1, 2], Haoqing Su[1, 2], Wentao Zhang[1, 2], Atsu Kludze[1, 2], Yuze Zheng[1, 2, 3], Chengxing He[1, 2], Rito Yanagi[1, 2], and Shu Hu*[1, 2]

[1] *Department of Chemical and Environmental Engineering, School of Engineering and Applied Sciences, Yale University, New Haven, CT 06520, USA.*

[2] *Energy Sciences Institute, Yale West Campus, West Haven, CT 06516, USA.*

[3] *College of Chemistry and Molecular Engineering, Peking University, Beijing 100871, China*

* Corresponding author: shu.hu@yale.edu

## Supplementary Note 1: Derivation of Equation (1)

In this analytic calculation, for simplicity, we assume that $H^+$ ions are generated uniformly on the anode surface and then diffuse into the bulk solution. We start by assuming $[H^+]$ in bulk seawater solution is zero. Furthermore, reactions involving $H^+$ with other species were intentionally omitted in order to emphasize the interplay between convection and diffusion mechanisms. Therefore, within the scope of this calculation, the $H^+$ produced at the anode is considered to be non-consumable. The concentration of a specific chemical species $i$, denoted as $C_i$, were calculated using multi-physics simulations. Equations S(1) and S(2) were used to describe the reactive transport of reactive species $i$, such as $CO_2$(aq), $H^+$, $OH^-$, $HCO_3^-$, and $CO_3^{2-}$:

$$\frac{\partial C_i}{\partial t} = -\nabla \cdot N_i + R_i \quad (2)$$

$$N_i = -D_i \nabla C_i + \boldsymbol{v} C_i - \frac{Z_i F}{RT} D_i C_i \nabla \phi \quad (3)$$

, where $C_i$ represents the concentration of species $i$, $N_i$ is the flux, $-\nabla \cdot N_i$ is the net influx (with the divergence of the flux is taken as a negative value), and $R_i$ is the net rate of production from chemical reactions per unit time, $D_i$ is the diffusion coefficient of species $i$, and $\boldsymbol{v}$ represents the local velocity. The reactive transport of seawater DIC species (e.g., $CO_2$(aq), $HCO_3^-$, and $CO_3^{2-}$), protons, and hydroxides were analyzed to determine whether a species is being produced ($R_i > 0$) or consumed ($R_i < 0$) in a chemical reaction. Using $R_{CO2}$ as an example, details of $R_i$ for are provided in the supporting information. According to Equation S(3), the Nernst-Planck equation, $N_i$ considers contributions from diffusion, convection, and a negligible migration flux. The boundary conditions, kinetic rate constants, diffusion coefficients, and electric charge of species are shown in Fig. S24b, tables S2 and S4.The boundary layer is defined as the envelope where $c > 0$, and the thickness of the boundary layer is denoted as $\delta(x)$ Equation S(4).

$$c(x,z) = c_0(x)\left(1 - \frac{z}{\delta(x)}\right) \quad (4)$$

This model is constrained by two boundary conditions: proton generation and their

transport by convection and diffusion. We assume a uniform generation rate of $H^+$ from seawater oxidation as described in Equation S(5). Under the assumption of non-consumptive $H^+$, the total flux of $H^+$ transported by convection through the cross-sectional area at $x = x_0$ is equivalent to the total amount of $H^+$ produced at the anode in the area at $x < x_0$ (Equation S6). Furthermore, the electrolyte flow between the two parallel plates conforms to Poiseuille flow, as described in Equations S(7), where $u_0$, $k$, $z$, and $d$ are the average flow velocity among the reactor, velocity gradient, the height above the anode, and thickness of the electrolyte (schematic in Fig. S24a), respectively.

$$-D\frac{\partial c}{\partial z}|_{z=0} = \frac{i}{F} \quad (5)$$

$$\int_0^{\delta(x)} c\,(x_0, z)u(z)\,\mathrm{d}z = \frac{i}{F}x_0 \quad (6)$$

$$u(z) = 6u_0 \cdot \left(-\left(\frac{z}{d}\right)^2 + \frac{z}{d}\right) \approx kz\ \left(k = 6\frac{u_0}{d}\right) \quad (7)$$

Equations S(4) to S(7) are combined to solve the thicknesses of the $H^+$ boundary layer as a function of the flow velocity $u_0$ and flow distance $x_0$ (Fig. 5a).

$$\delta \approx \left(\frac{6Dx_0}{k}\right)^{\frac{1}{3}} = \left(\frac{x_0Dd}{u_0}\right)^{\frac{1}{3}}$$

Employing this semi-quantitative model, we further predict the spatial distribution of the additional $CO_2$ flux generated by acidification, as described by following Equation. This provides a rationale for the observed "nose-shaped" profile of the flux distribution in Figure 6b.

**The following is an expression for convective flux**

$$\text{Flux} = u \cdot c = k \cdot c_0(x)z\left(1 - \frac{z}{\delta(x)}\right)$$

## Supplementary Note 2: COMSOL simulations

**Model configuration:**

We modeled our PEC reactor as a flatbed device, with an upstream photoanode and downstream photocathode arranged side-by-side. The modeling was conducted in 2D as the electrode behavior across the width dimension is uniform. Both the anode and cathode were 1 cm long and 2 cm apart at the bottom of the seawater chamber, with the electrolyte above the electrodes 1 mm in height (Fig. 5a). These dimensions exactly match the dimensions of the 3D printed vortex reactor used in the aforementioned experiments (Fig. 2a). Electrolyte flow velocity in our model was determined from our experimental data, with the chamber walls modeled with no-slip boundary conditions. The Reynolds numbers at flow velocities of 0.16 m/s, 0.34 m/s, 0.56 m/s, and 0.77 m/s are 320, 680, 1120, and 1540 respectively, all below 2000, confirming that our vortex reactor operates under laminar flow conditions.

**COMSOL model validation by analytical boundary calculations:**

The COMSOL simulated $CO_2$ concentration profile shows that the $CO_2$ concentration on the anode surface is the highest, at 0.43 mol/m$^3$, and the $CO_2$ concentration decreases as the distance from the anode increases. When it decreases to 5% (0.02 mol/m$^3$) of the surface concentration, we believe it is close to the bulk $CO_2$ concentration and has no significant contribution to downstream $CO_2$ reduction. The boundary layer calculated by the analytic model, i.e., Equation (1) is consistent with the trend of $CO_2$ concentration of 0.02 mol/m$^3$, indicating the accuracy of the fluid mechanics calculations and COMSOL simulations.

**COMSOL model validation by analytical boundary calculations:**

Da is defined as the ratio of the flow transport timescale from the anode to the cathode, to the chemical lifetime of *in situ* generated $CO_2$(aq). The convective flow timescale is given by L/$u$, where L is the distance between the anode and cathode, and $u$ represents the average flow velocity of the boundary layer flow within the boundary layer at X =

1 cm (Fig.6a). The chemical life of $CO_2$ is determined as follows. In our COMSOL simulations, we introduce a pulse of $OH^-$ into a static buffer solution with a pH of 4 (a typical pH value at the anode surface in static electrolytes). This pulse causes the pH of the buffer to eventually drop to 8.3, the pH of static seawater. We measure the time required for the $CO_2$ concentration to decay to 1/e of its initial value following the introduction of an $OH^-$ pulse. This decay time is defined as the chemical lifetime of *in situ* generated $CO_2$(aq).

**Quantitative boundary layer analysis for $H^+$ and $CO_2$(aq):**

Our COMSOL Multiphysics simulations indicated that the concentrations of various species gradually change from the electrode surface to the seawater bulk. To calculate the thickness of the boundary layer of each relevant chemical species obtained from COMSOL Multiphysics simulation results, the following equation was used:

$$\frac{[i]_{z}-[i]_{\text{bulk}}}{[i]_{\text{surface}}-[i]_{\text{bulk}}} \times 100\% = 1\%$$

Taking $H^+$ for example, the $[H^+]_{bulk}$ is the $H^+$ concentration of bulk solution ($5.2 \times 10^{-9}$ mol/L), $[H^+]_{surface}$ is the $H^+$ concentration of electrode surface, and $[H^+]_z$ is the $H^+$ concentration within the boundary layer. When the value calculated by the above Equation is 1%, the proton concentration at that position is much smaller than the proton concentration at the electrode surface. Considering $[H^+]_{bulk} = [H^+]_{z=100\mu m}$, and $[H^+]=10^{-pH}$, we extracted the thickness of the $H^+$ boundary layer at different flow rates using the pH map (Fig. S26a). At flow rates of 0.16, 0.34, 0.56, and 0.77 m/s, the boundary layer thicknesses were 67, 65, 57, and 56 μm, respectively. It can be observed that the $H^+$ boundary layer thickness decreases as the flow rate increases. The values agree with the trend of COSOL simulations though consistently higher due to overestimation of measured surface pH. Within the boundary layer, $HCO_3^-$ is acidified by $H^+$ to produce $CO_2$(aq). Outside the $H^+$ boundary layer, $[H^+]$ is too low for us to consider the effective acidification of $HCO_3$- to generate $CO_2$. Hence, this position is defined as the boundary of shear flow transport. The boundary layers for $H^+$ and $CO_2$(aq) are shown in Fig. S26.

**Reactive transport equation for $CO_2$(aq):**

$$\frac{\partial C_{CO_2}}{\partial t} = \nabla \cdot \left(D_{CO_2} \nabla C_{CO_2}\right) - \nabla \cdot \left(\boldsymbol{v} C_{CO_2}\right) + \nabla \cdot \left(\frac{Z_{CO_2} F}{RT} D_{CO_2} C_{CO_2} \nabla \phi\right) - k_{f1} C_{CO_2} + k_{r1} C_{HCO_3^-} C_{H^+} + k_{r2} C_{HCO_3^-} - k_{f2} C_{CO_2} C_{OH^-}$$

**Analysis of COMSOL numerical simulations for $CO_2$(aq) flux:**

The diffusion length (L), influenced by the transport time ($L = \sqrt{Dt}$), decreases with the increasing flow velocity, leads to a reduction in the z-direction diffusion of $CO_2$(aq). Compared to the $CO_2$(aq) flux of 7.5 $mmol{\cdot}m^{-2}{\cdot}s^{-1}$ (Fig. S39) at 0.16 m/s at the front end of cathode, a $CO_2$(aq) flux of 13.53 $mmol{\cdot}m^{-2}{\cdot}s^{-1}$ was achieved at 0.77m/s, indicating that an elevated flow rate corresponds to higher $CO_2$(aq) flux near the cathode surface. This can explain why the CO selectivity and STF efficiency increases at higher flow velocities (Fig. 3a), with an optimized flow velocity allows for more $CO_2$(aq) to be transported to the cathode surface, leading to higher $CO_2$ coverage and CO Faradaic efficiencies.

**Performance, selectivity, and stability of Si photocathode under 1-atm $CO_2$ purge:**

Although $CO_2$ extraction from seawater using a $BiVO_4$ photoanode is feasible, the conversion of $CO_2$ into value-added products remains challenging due to the inherent issues of low stability and selectivity associated with Si photocathodes. To protect Si from corrosion in the electrolyte, a commonly used strategy is to apply a $TiO_2$ protection on the surface of Si to isolate the Si and electrolyte. However, the $TiO_2$/Si interface tends to generate $H_2$, resulting in low $CO_2$ conversion selectivity[1]. Moreover, if a metal layer were selected as the protective layer, it would impede the transmission of light and result in a reduction in the current density of the Si photocathode[2].

To overcome these obstacles, a low-cost method was developed to fabricate Si photocathodes (Fig. S4). We can scale up this manufacturing approach to make photoelectrodes covering a large area of ocean surface. To enhance the driving force for PEC $CO_2$R, an amorphous-Si (*a*-Si) passivated monocrystalline Si photocathode with a back-illuminated configuration was constructed (Fig. S37). The Si surface was textured with micro-pyramids to decrease surface reflection (Fig. S3b). The dangling bonds on Si surface were fixed by *a*-Si, achieving ultra-low surface defects density and long minority carrier diffusion length (longer than ~2000 μm). Therefore, the light absorption and surface reaction can be separated on different side of Si substrate (thickness in 150 μm), the photogenerated minority carriers can transfer from the bulk to the surface to participate in the $CO_2$ conversion reaction before recombination. In a back-illuminated configuration, the catalytic layers will not compete with Si for light absorption. To suppress the $H_2$ production, a 20 μm thick layer of Ag paste was uniformly scribbled to the surface of Si substrate in direct contact with the indium tin oxide (ITO) layer (Fig. S38), which serves as the catalytic and protective layer for Si photocathodes. It is worth noting that the preparation method is easily scalable and possesses low costs as it does not require the use of a vacuum for the fabrication of the protective and catalytic layer. For PEC measurements, the Si substrate was encapsulated as electrodes without further treatment. Light-driven $CO_2$ reduction on the photocathode was performed under AM 1.5G illumination (100 mW/cm$^2$) in sea water with $CO_2$ purge (pH 7) under ambient conditions. The Si photocathode with single junction exhibits an onset potential of 0.3 V vs RHE (defined as the potential required to achieve a photocurrent of 0.1 mA/cm$^2$) (Fig. 1d). Although the Si photocathodes have shown promising onset potential and ABPE (Fig. S9), the interaction point between the Si photocathode and $BiVO_4$ photoanode remains relatively low, leading to suboptimal unbiased overall performance. It should be noted that as the potential negatively increases the reductive current density increases (Fig. S4e), whereas the FE of CO is decreased. This can be attributed to low bicarbonate concentration present in sea water, which limits the ability to sustain higher $CO_2$ reduction current densities at more negative potentials. Hence, $H_2$ production becomes the domain reaction at a higher potential.

# Supplementary Figures

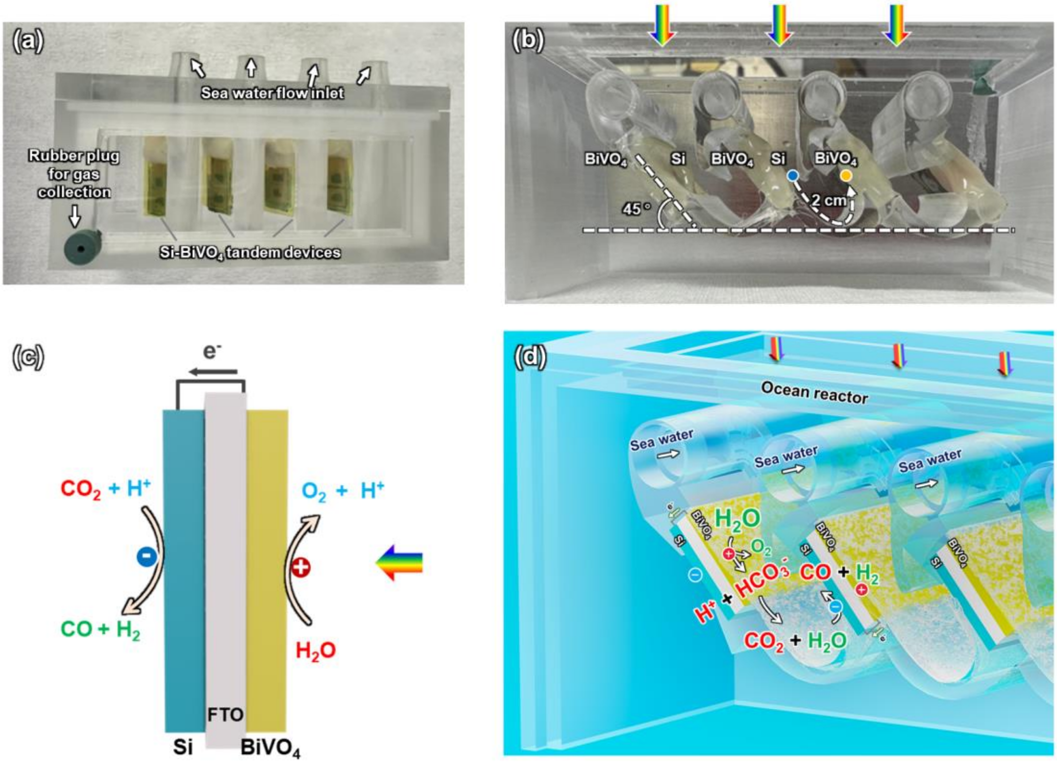


**Fig. S1** Photographs of the (a) top and (b) cross-sectional view of flowing seawater reactor utilized in this study. (c) Schematic of $BiVO_4$/Si tandem device and corresponding charges transfer. (d) Schematic of charges transfer between each tandem devices in photoreactor.

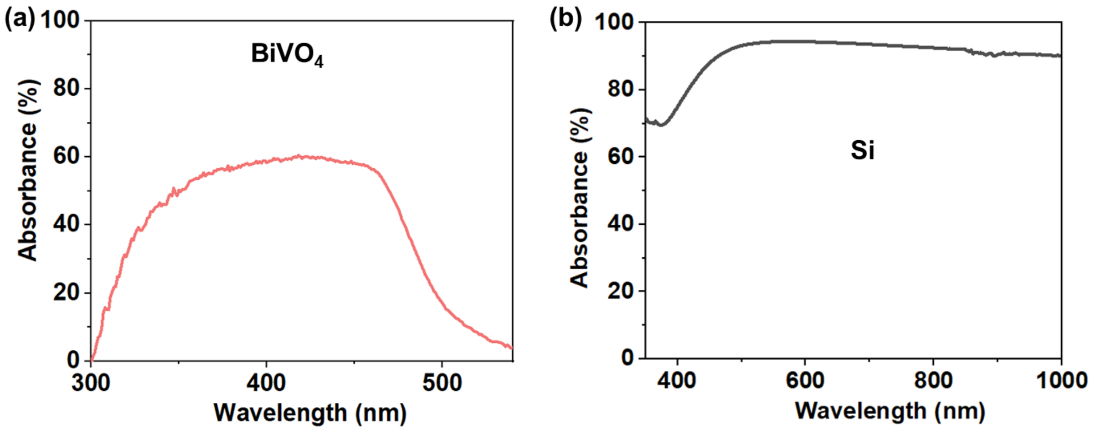


**Fig. S2** Light absorbance of (a) $BiVO_4$ photoanode and (b) Si photocathode.

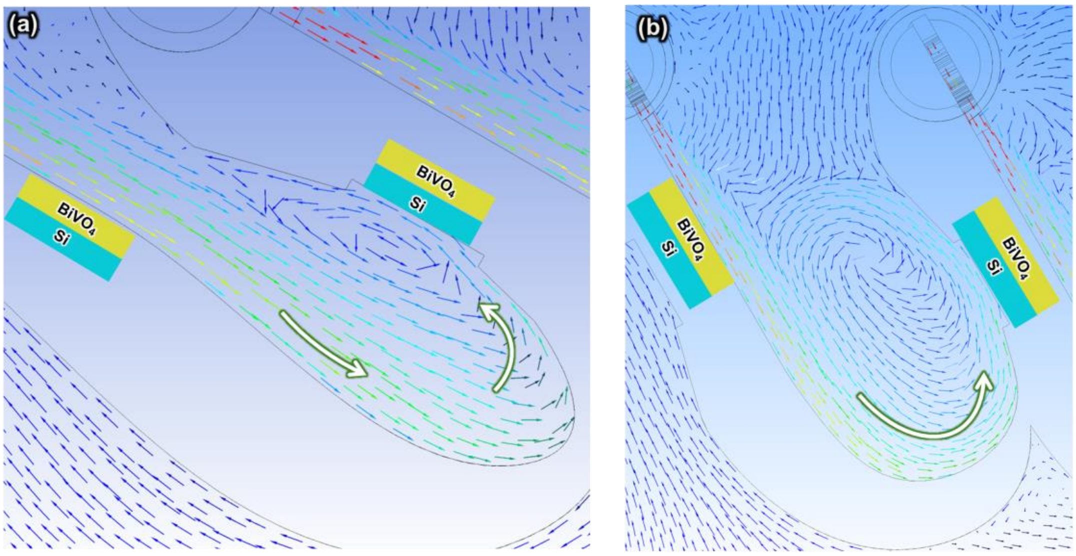


**Fig. S3** The flow field simulations for representative tilt angles of 30° (a) and 60° (b).

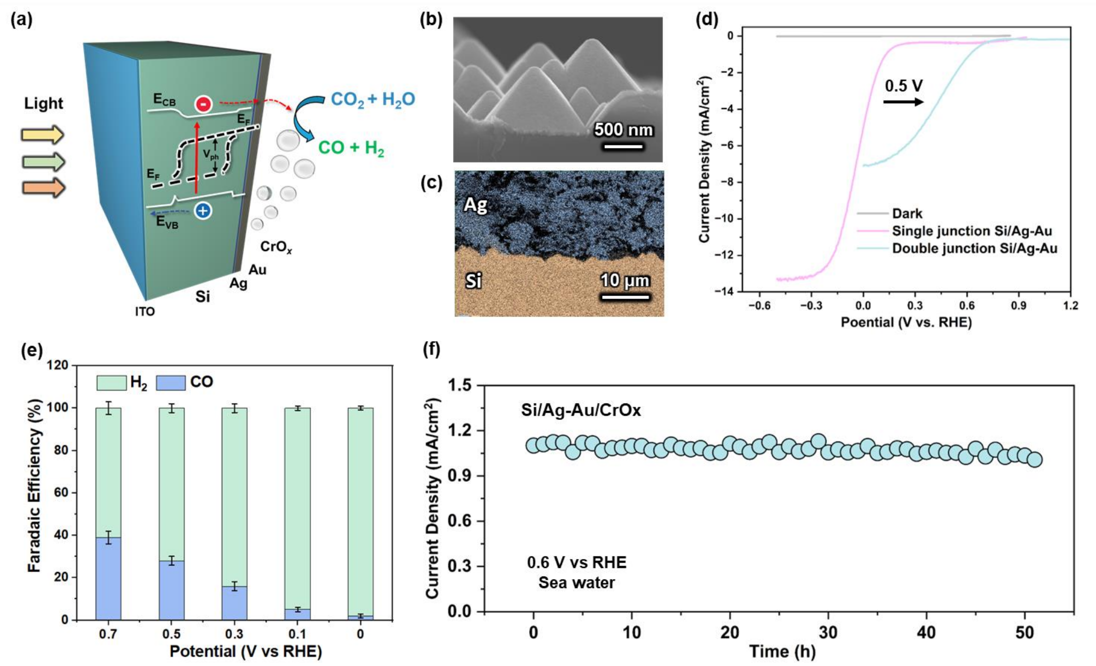


**Fig. S4 Si photocathodes for in-situ generated $CO_2$ conversion in seawater.** a, The schematic energy band diagram of Si photocathodes under back illumination configuration. The cross-section SEM image (b) and corresponding element mapping (c) of Si photocathodes. d, J-V curves of Si photocathodes with single and double junction, e, Potential dependent faradaic efficiencies toward CO and $H_2$. f, stability of double Si photocathode at 0.6 V vs RHE.

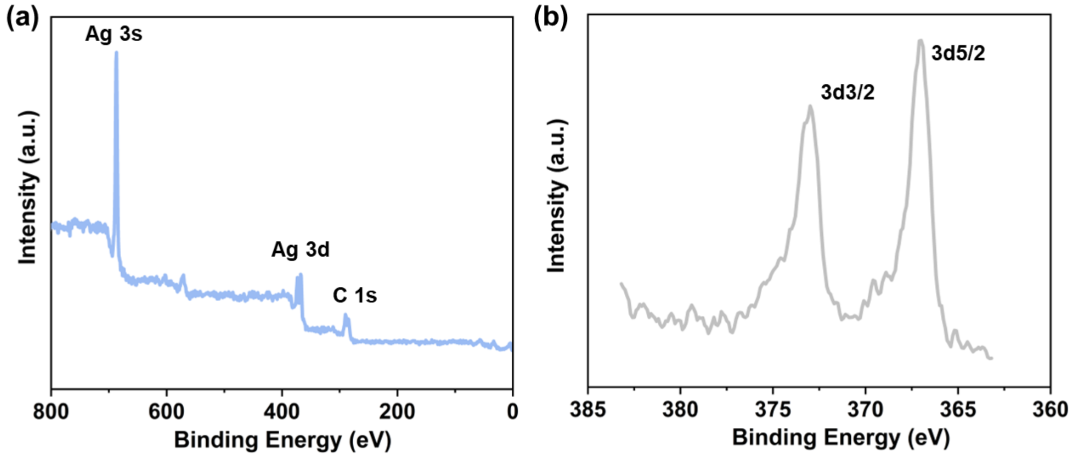


**Fig. S5** XPS of Si photocathode. (a) XPS survey spectrum. (b) High-resolution XPS spectra of the Ag 3d.

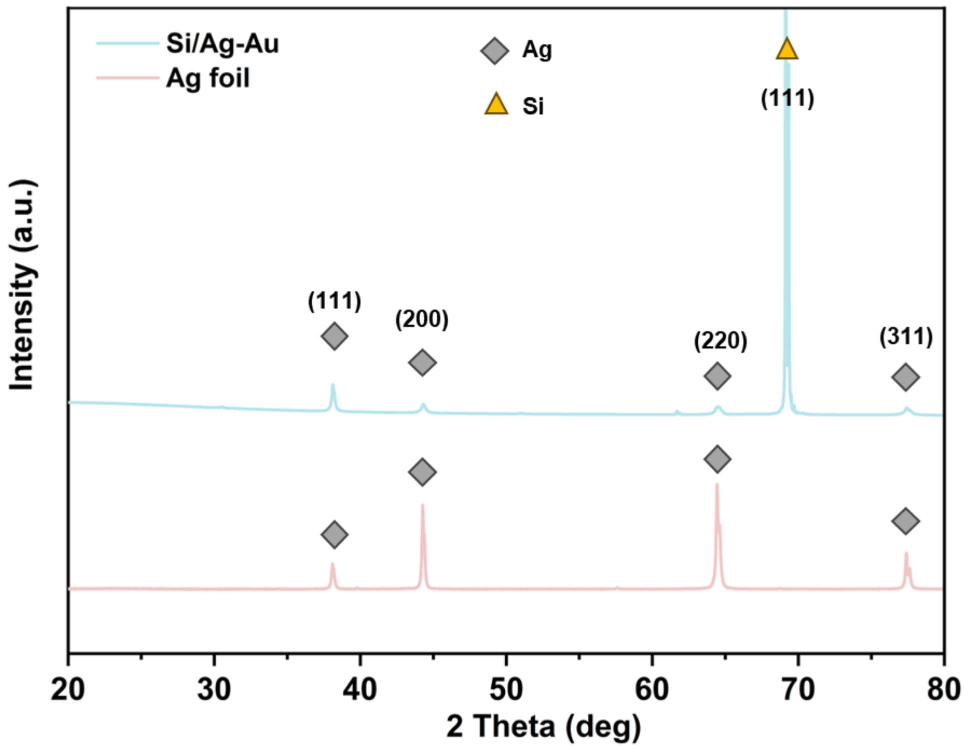

**Fig. S6** XRD patterns of Si photocathode and Ag foil.

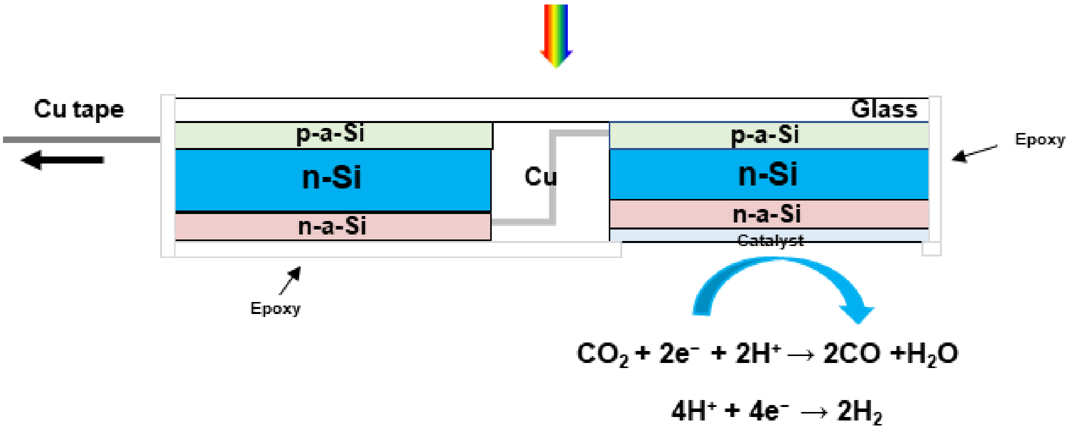


**Fig. S7** Schematic of double junction Si photocathode for seawater $CO_2RR$

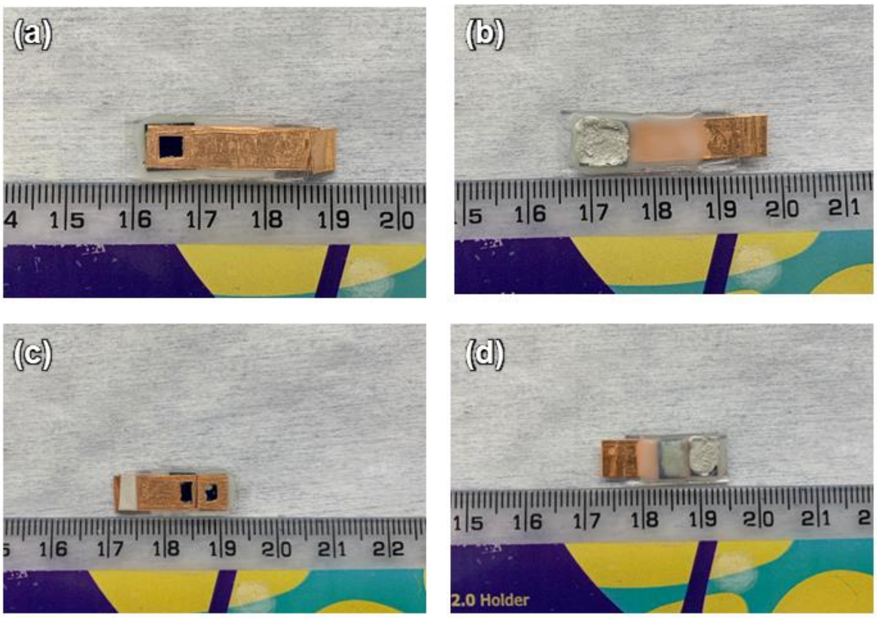


**Fig. S8** Photograph of back-illuminated Si photocathodes. (a) light-facing side and (b) surface reaction side of single junction Si. (c) light-facing side and (d) surface reaction side of double junction Si photocathodes.

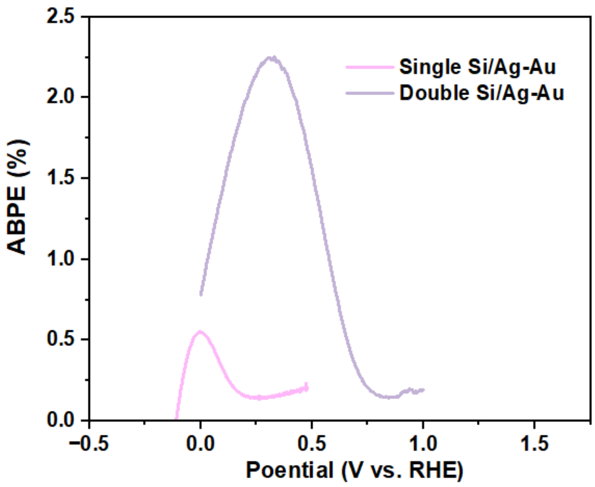


**Fig. S9** ABPEs of single and double Si photocathodes.

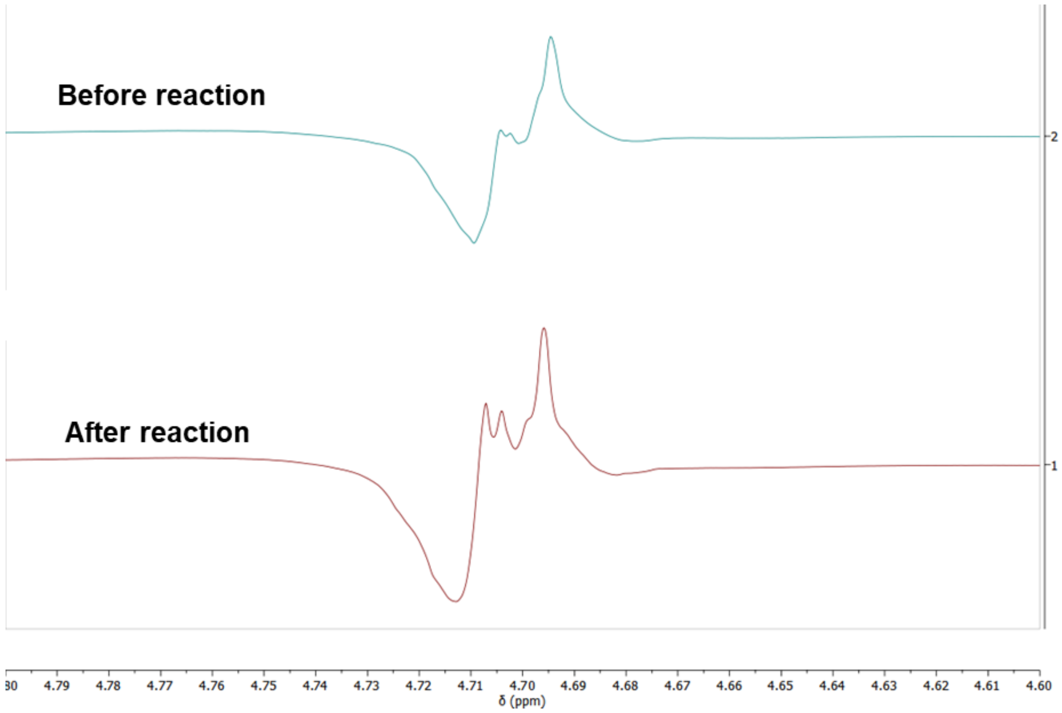


**Fig. S10** NMRs of sea water before and after PEC $CO_2R$.

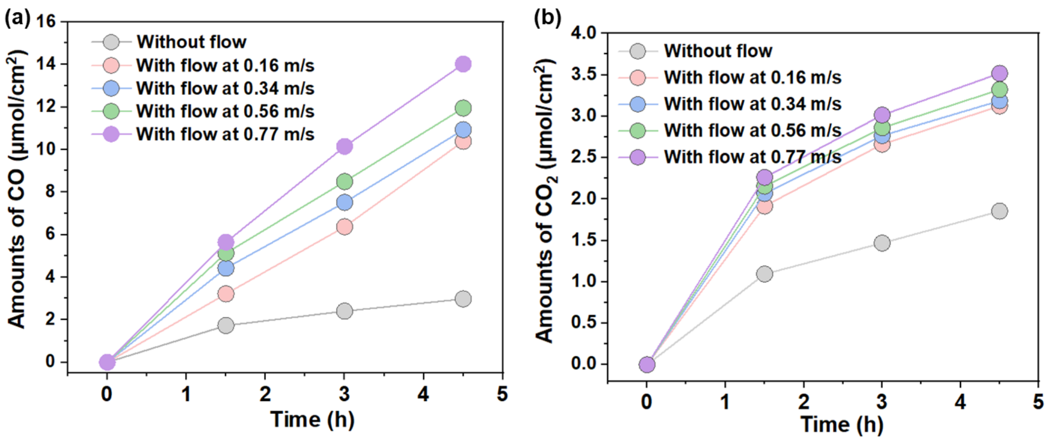


**Fig. S11** (a) CO gas production and (b) $CO_2$ extraction over time at varying flow velocities under AM 1.5G 1-sun illumination (100 mW/cm$^2$).

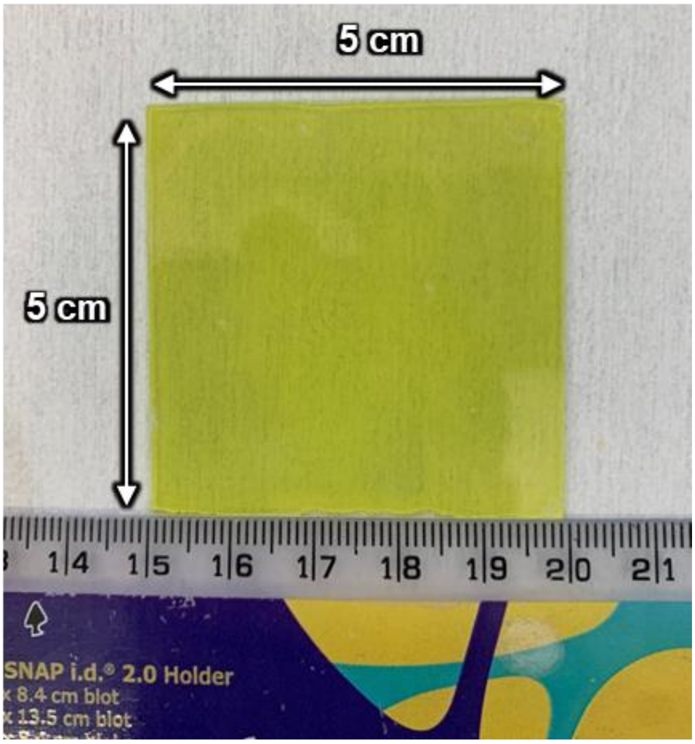

**Fig. S12** Photograph of $BiVO_4$ photoanode.

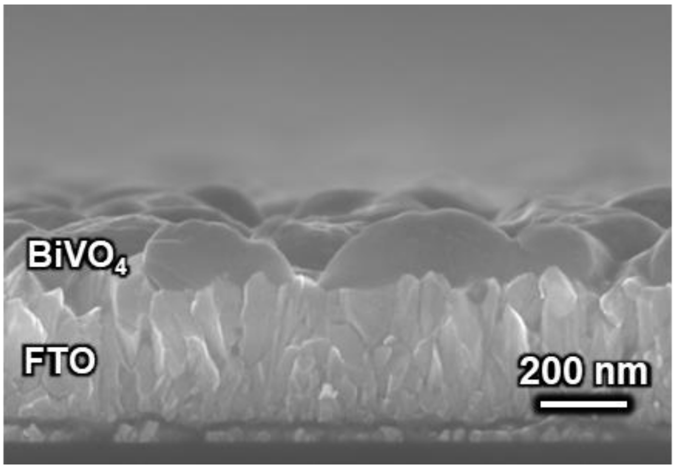


**Fig. S13** SEM image of $BiVO_4$ photoanode cross section.

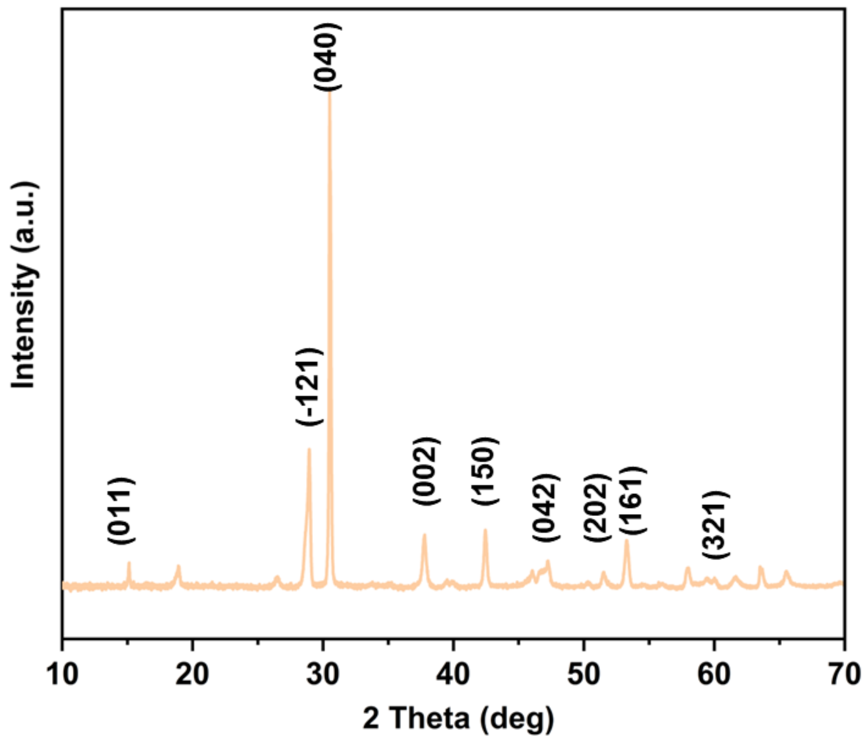


**Fig. S14** XRD pattern of $BiVO_4$ photoanode.

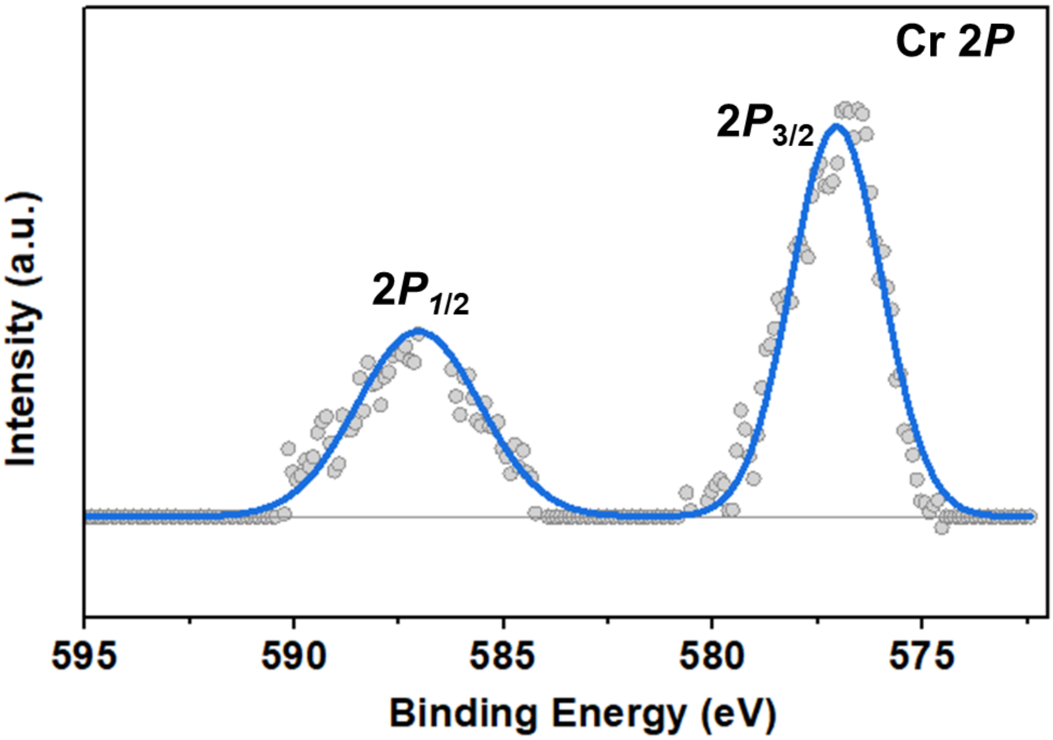


**Fig. S15** XPS of Cr 2p for $BiVO_4$/NiFe(OH)$_x$/Cr$O_x$ photoanode.

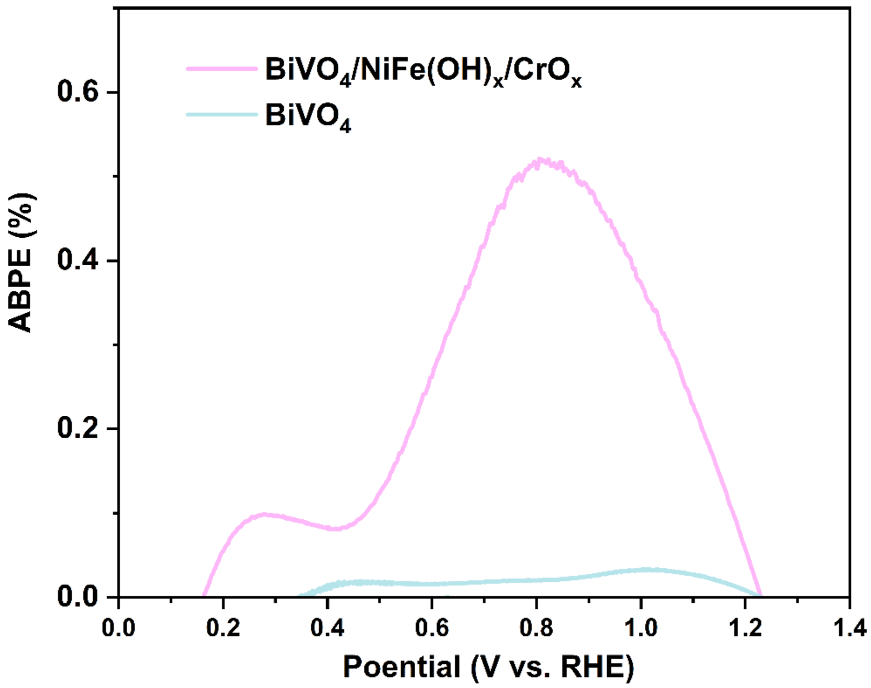


**Fig. S16** ABPEs of $BiVO_4$ photoanode with and without catalyst at varying applied potentials.

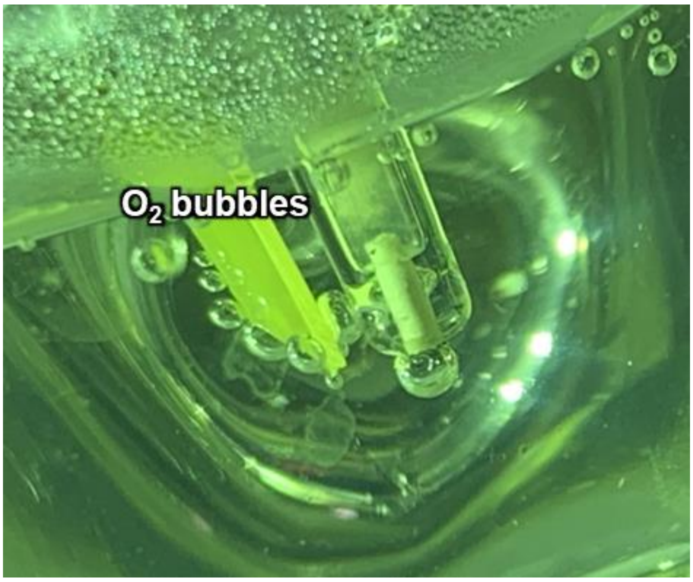


**Fig. S17** Photograph of $BiVO_4$ photoanode during seawater oxidation.

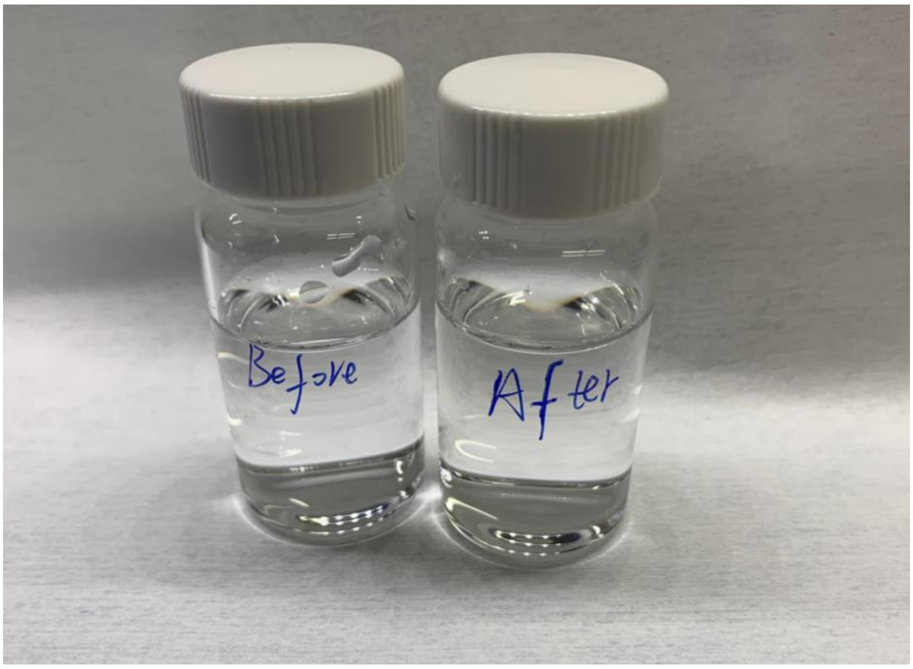


**Fig. S18** Photograph of seawater solution with excessive iodide ions added, taken before and after 20 hours of seawater oxidation.

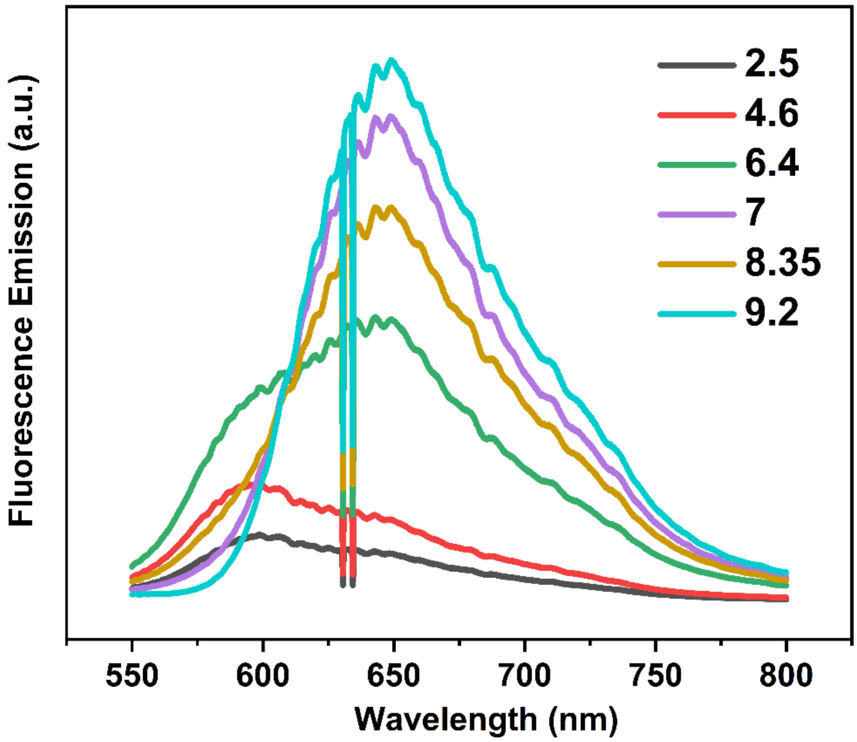


**Fig. S19** Raman spectrums of Carboxy SNARF-1 in electrolyte at varying pH.

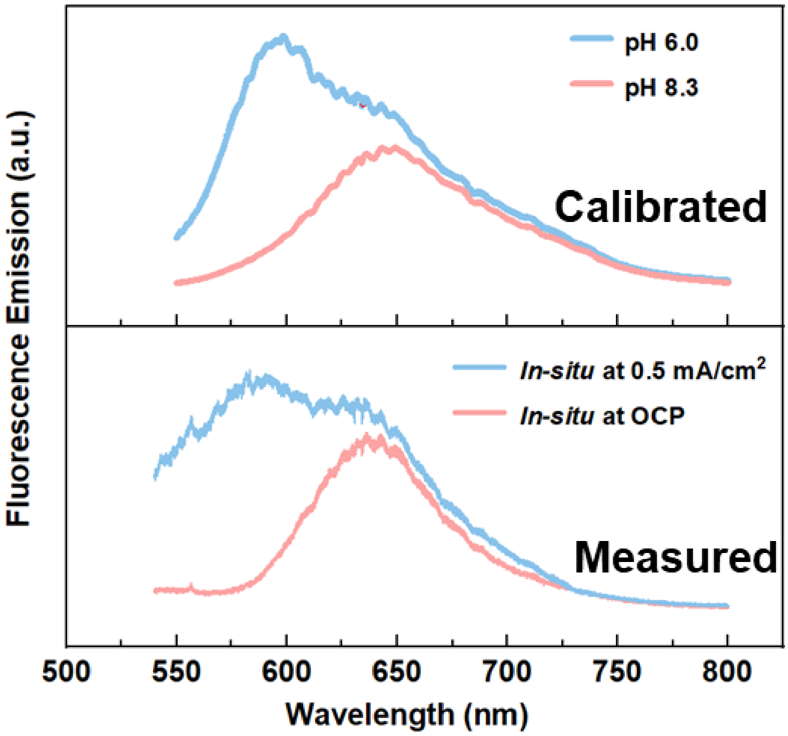


**Fig. S20** The fluorescence emission spectra curves of calibrated (upper) and measured (lower) pH.

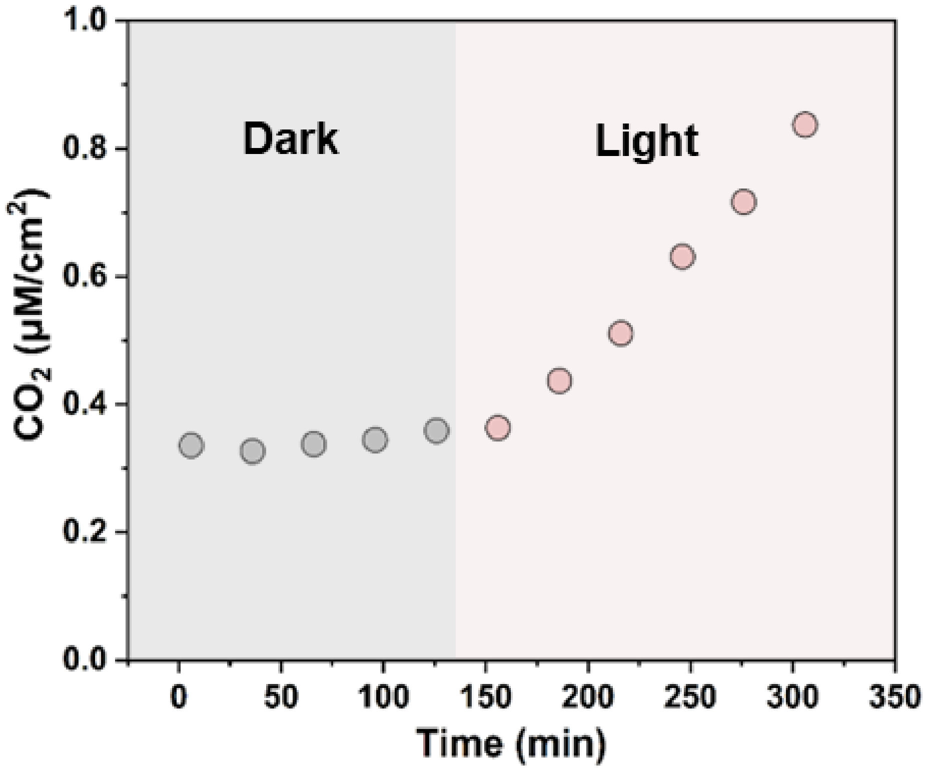


**Fig. S21** $CO_2$ gas extraction over time.

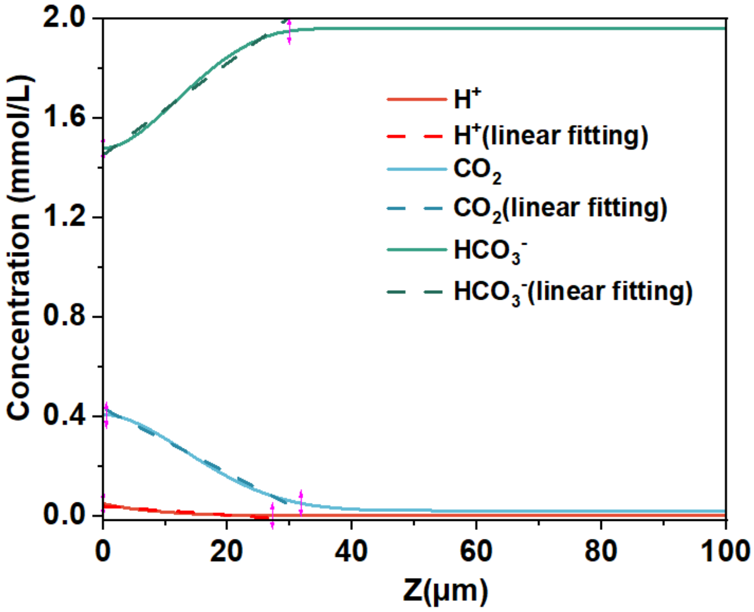


**Fig. S22** The spatial concentration profiles of $H^+$, $CO_2$, $HCO_3^-$ species in the z direction obtained using COMSOL Multiphysics simulation.

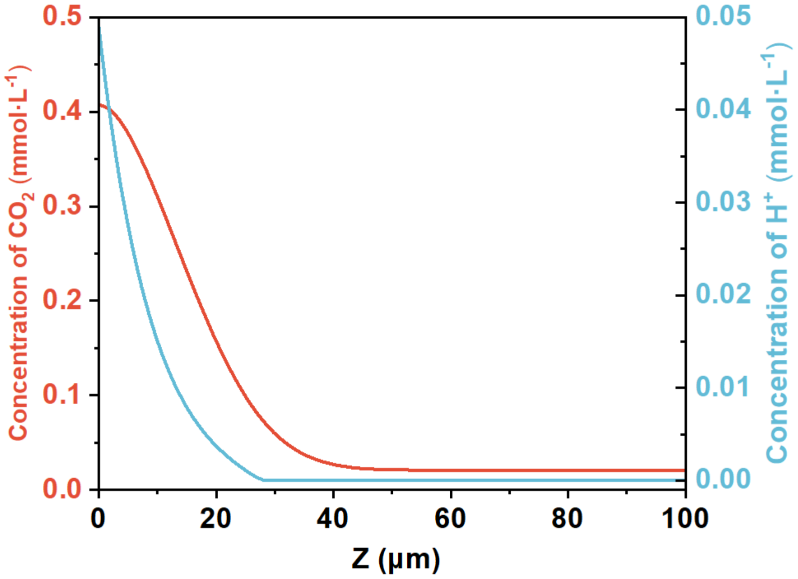


**Fig. S23** Concentration profiles of $CO_2$ and $H^+$ at x = 0.8 cm at a flow rate of 0.77m/s.

(a)

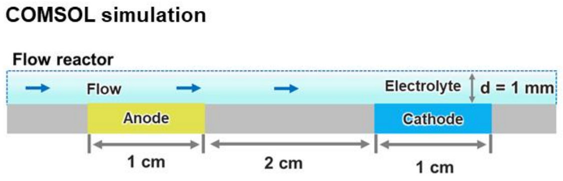


(b)

$$H^+ + OH^- \underset{f_w}{\overset{r_w}{\rightleftharpoons}} H_2O$$

$$CO_2 + H_2O \underset{r_1}{\overset{f_1}{\rightleftharpoons}} HCO_3^- + H^+$$

$$HCO_3^- \underset{f_3}{\overset{r_3}{\rightleftharpoons}} CO_3^{2-} + H^+$$

$$HCO_3^- \underset{f_2}{\overset{r_2}{\rightleftharpoons}} CO_2 + OH^-$$

$$HCO_3^- + OH^- \underset{f_4}{\overset{r_4}{\rightleftharpoons}} CO_3^{2-} + H_2O$$

The equation for $CO_2$:

$$\frac{\partial C_{CO_2}}{\partial t} = \nabla \cdot \left(D_{CO_2} \nabla C_{CO_2}\right) - \nabla \cdot \left(\boldsymbol{v} C_{CO_2}\right) + \nabla \cdot \left(\frac{Z_{CO_2} F}{RT} D_{CO_2} C_{CO_2} \nabla \phi\right) - k_{f1} C_{CO_2} + k_{r1} C_{HCO_3^-} C_{H^+} + k_{r2} C_{HCO_3^-} - k_{f2} C_{CO_2} C_{OH^-}$$

**Fig. S24** (a) Schematic of the COMSOL numerical and numerical simulation (figure not to scale for clarity) and (b) kinetic rate laws for carbonate speciation in a seawater flow.

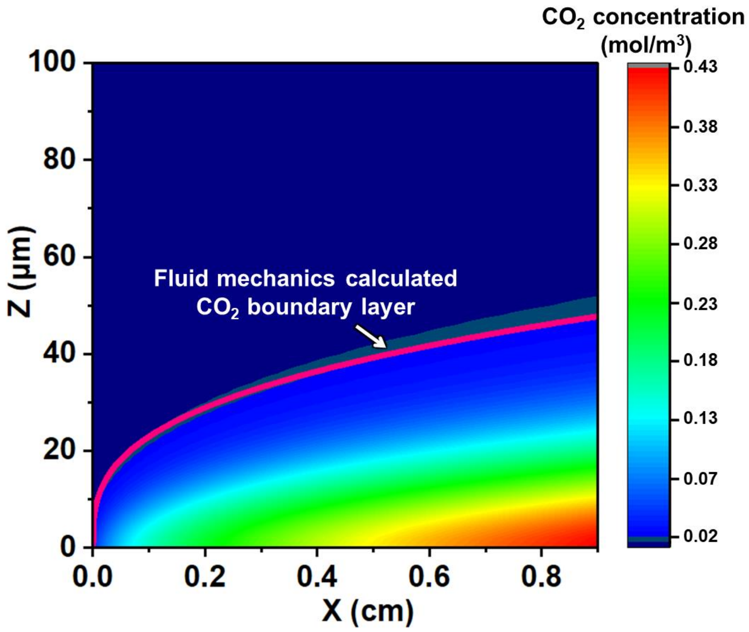


**Fig. S25** Comparison between fluid mechanics calculated $CO_2$ boundary layer (red line) and COMSOL simulated $CO_2$ concentration profile of anode at a flow velocity of 0.77 m/s.

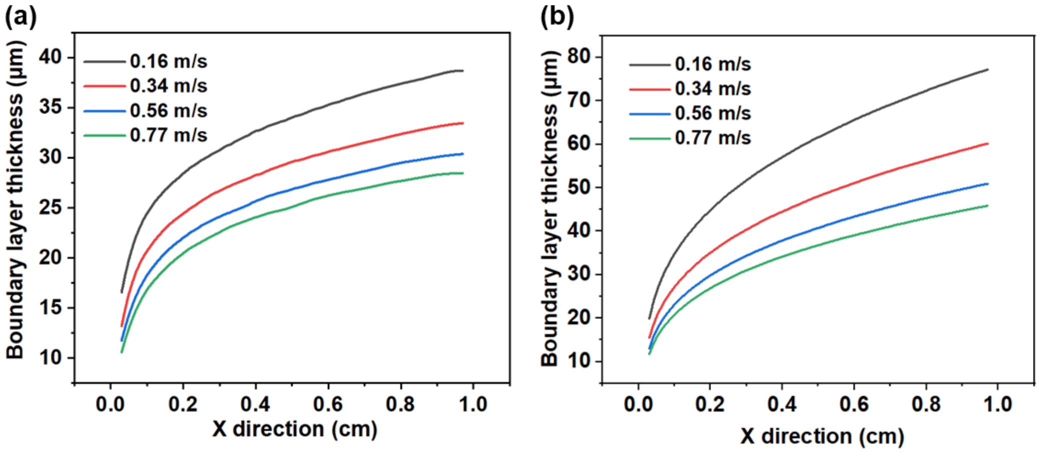


**Fig. S26** COMSOL simulated boundary layer thickness of (a) $H^+$ and (b) $CO_2$(aq) as a function of location along the flow stream. See "Quantitative boundary layer analysis" Section for the definition of boundary layers shown above.

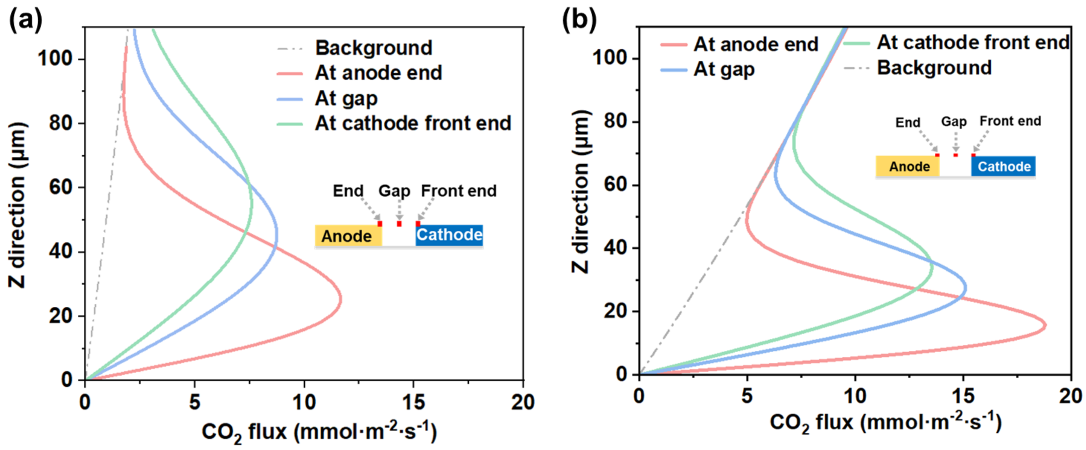


**Fig. S27** COMSOL simulated convective flux of $CO_2$(aq) at 0.16 m/s (a) and 0.77 m/s (b) at the end of the anode (X = 1 cm), at the gap between cathode and anode (X = 2 cm), and at the front end of the cathode (X = 3 cm).

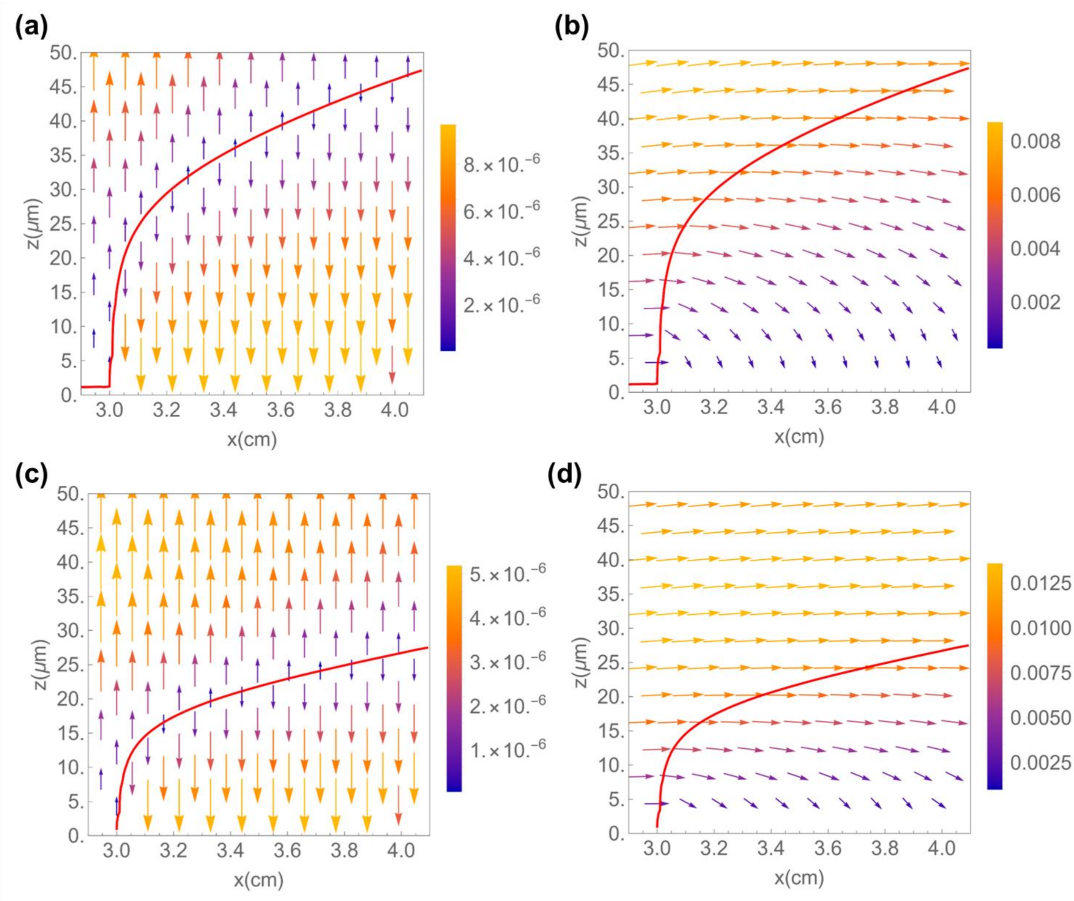


**Fig. S28** (a) The diffusion flux of $CO_2$(aq) 0.16 m/s. (b) The total diffusion flux of $CO_2$(aq) 0.16 m/s. at 0.16 m/s. (c) The diffusion flux of $CO_2$(aq) 0.77 m/s. (d) (b) The total diffusion flux of $CO_2$(aq) 0.77 m/s.

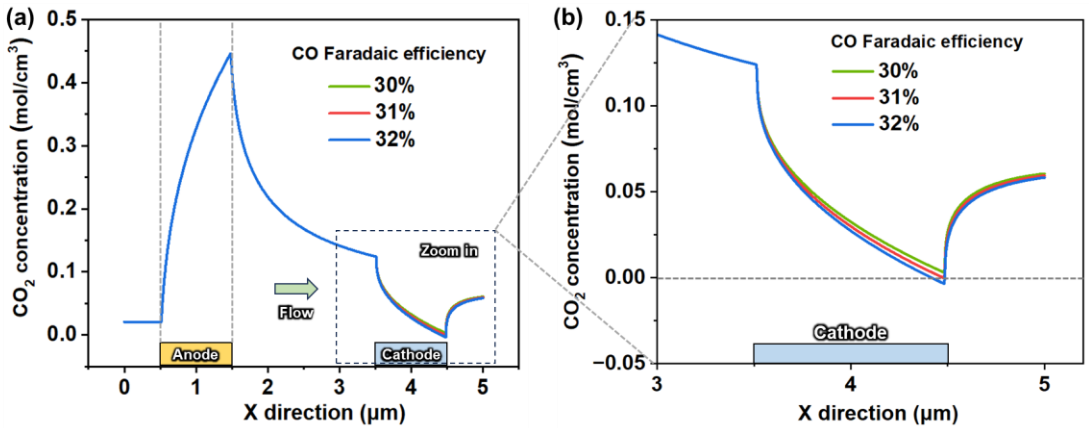


**Fig. S29** COMSOL Simulated maximum faradaic efficiency of CO for 2 cm gap configuration at the flow velocity of 0.77 m/s (a) and its zoom-in plot(b).

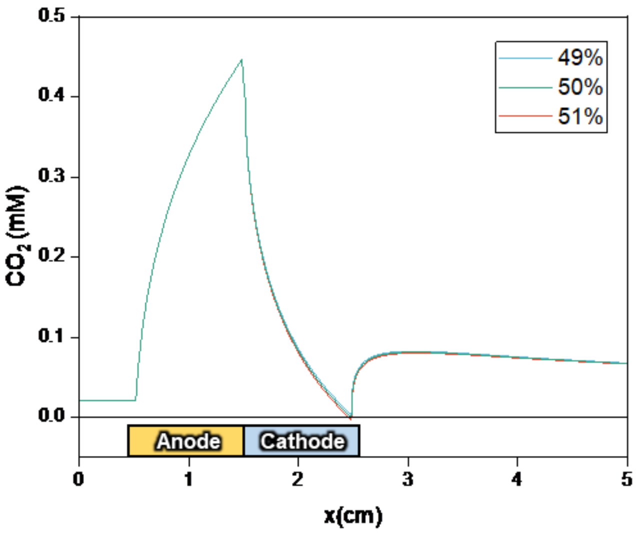


**Fig. S30** Simulated maximum faradaic efficiency of CO for 0 cm gap configuration at the flow velocity of 0.77 m/s.

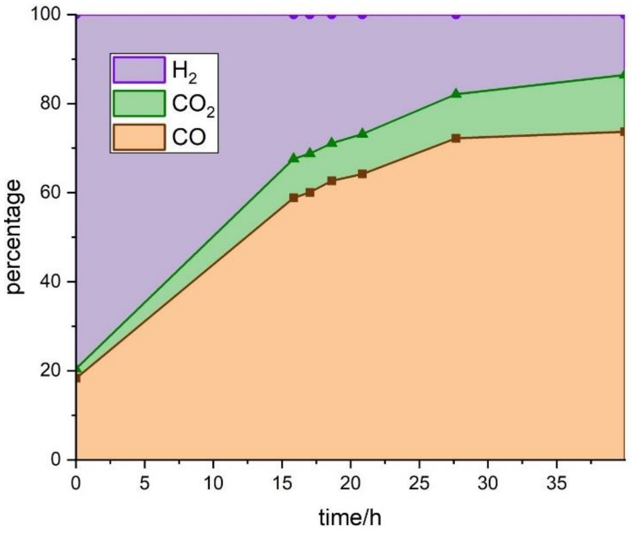


**Fig. S31** Time course of volume percentage of CO derived from selective (photo-)electrochemical $CO_2$ reduction in the same PEC flow device employing boundary flow with seawater and syngas recirculation, indicating the upper limit of the CO selectivity achievable during direct PEC $CO_2$R process in flowing seawater.

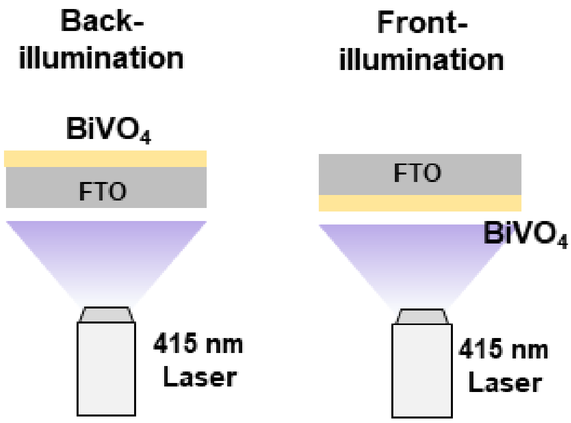


**Fig. S32** Schematic of back- and front-illumination configuration of $BiVO_4$ photoanode.

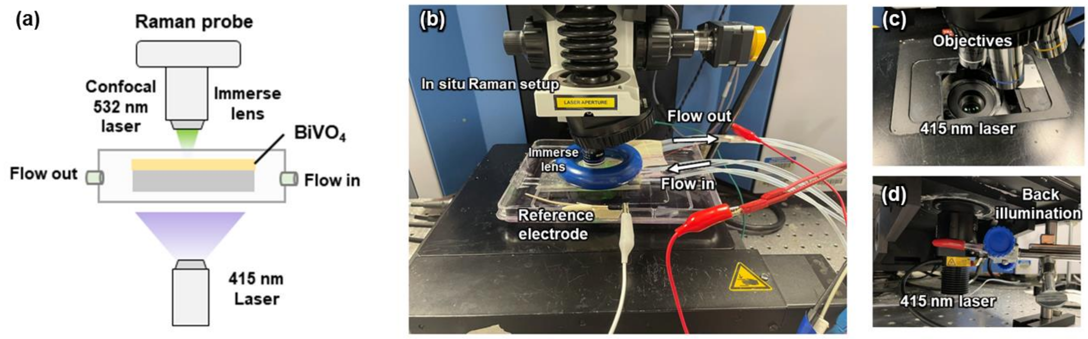


**Fig. S33** Schematic (a) and photograph (b, c, d) of customized flow device apparatus used for the in-situ fluorescence measurement in a fluid flow.

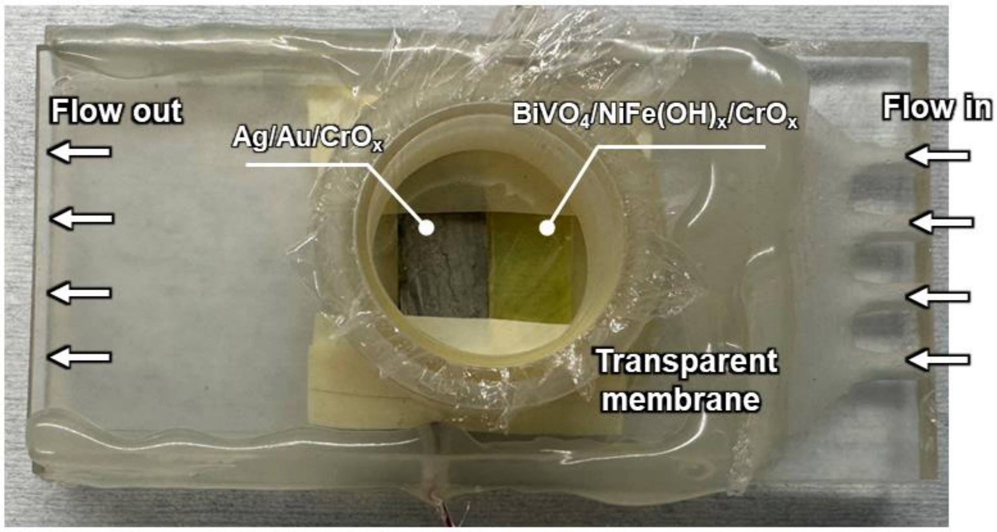


**Fig. S34** Photograph of the flow PEC device used during in-situ photo-fluorescence via confocal Raman spectroscopy. Blue light is illuminated from the back side, achieving 0.5 mA cm$^{-2}$ photocurrent density matching the PEC device measurements.

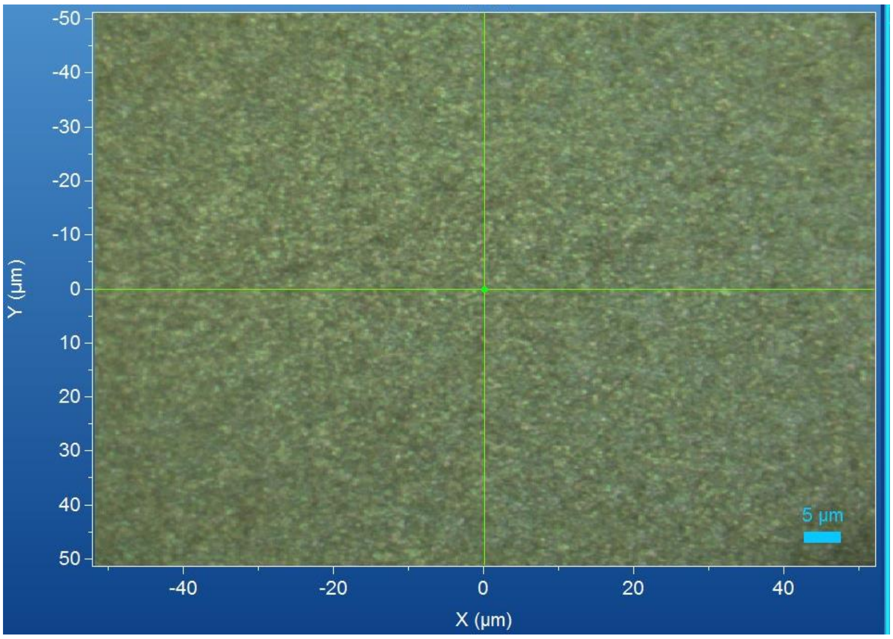

**Fig. S35** Image of $BiVO_4$ photoanode captured using Raman 60× immersion lens.

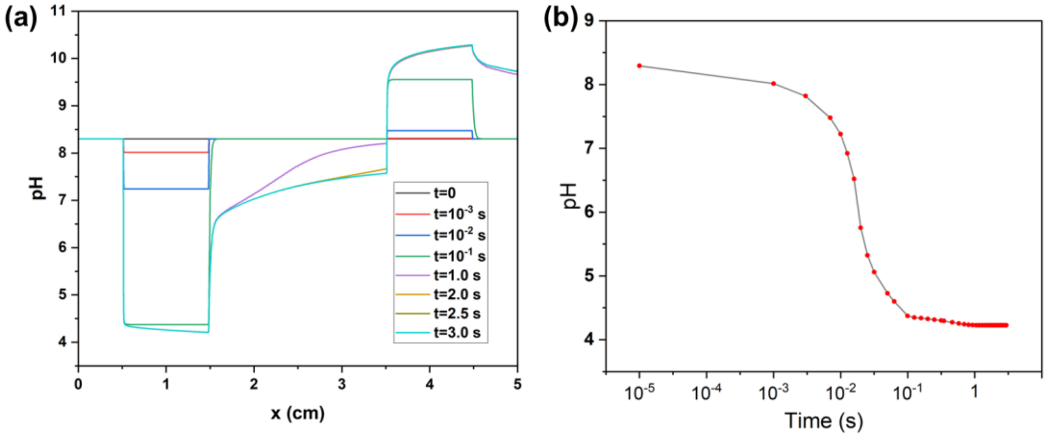


Fig. S36 COMSOL simulated time-dependent pH under velocity of 0.16 m/s at (a) cathode end and (b) z = 0 μm.

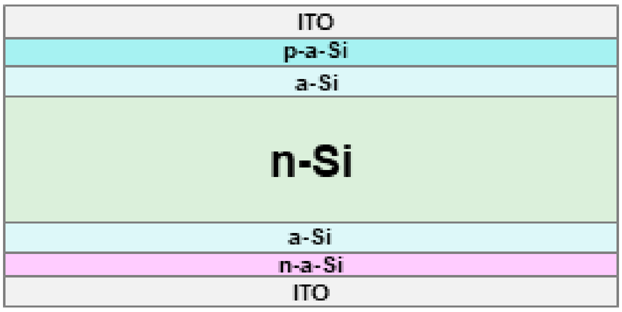


**Fig. S37** Schematic of Si photocathode with a-Si passivation layer (a-Si)

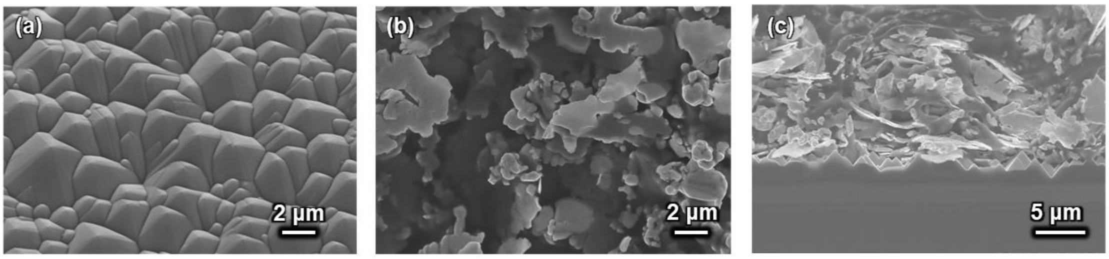


**Fig. S38** Top view SEM images of Si photocathode after texture (a) with Ag and Au layer (b). (c) cross-section SEM image of Si photocathode with Ag and Au layer.

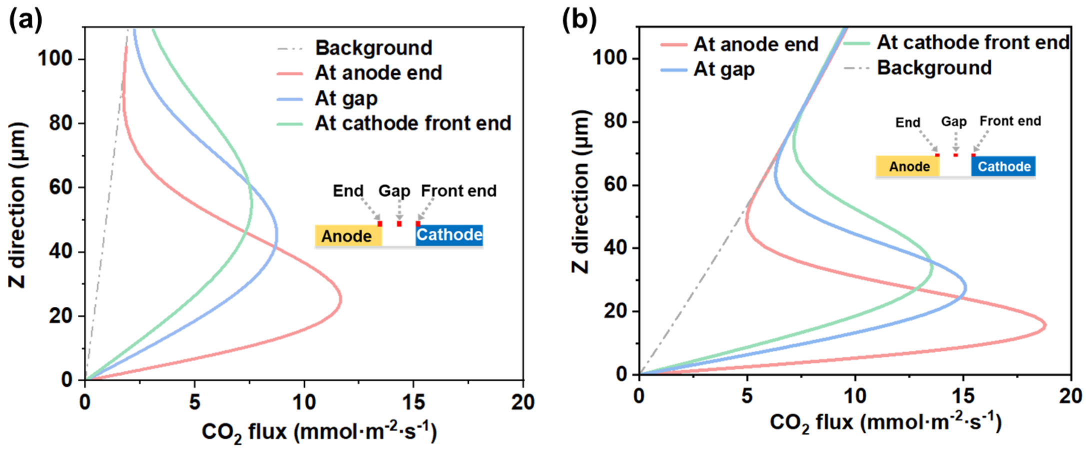


**Fig. S39** COMSOL simulated convective flux of $CO_2$(aq) at 0.16 m/s (a) and 0.77 m/s (b) at the end of the anode ($x_0$ = 1 cm), at the gap between cathode and anode, and at the front end of the cathode.

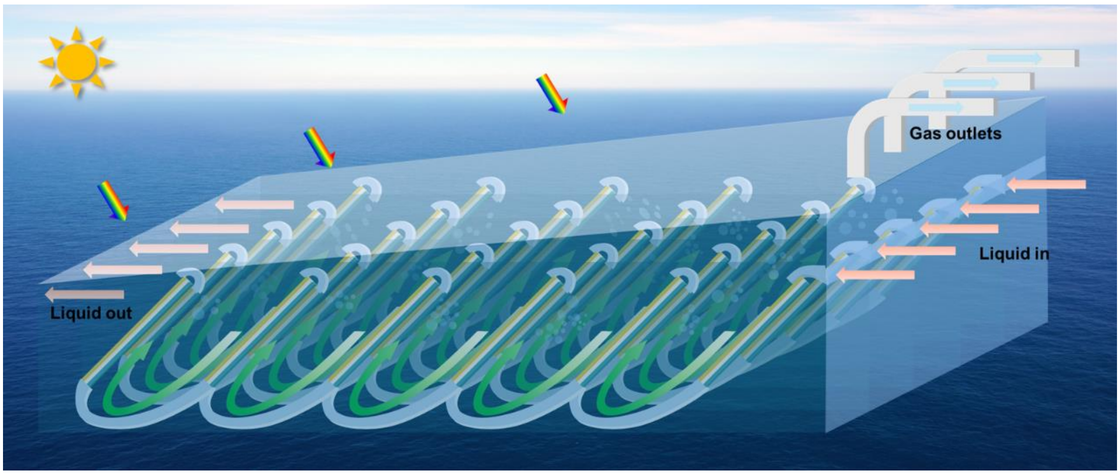


Fig. S40 Schematic diagram of gas collection for large-scale flow reactors floating on seawater.

**Table S1 Elemental composition of instant ocean water**

| Major elemental | Concentration (mmol/Kg) |
|---|---|
| $Na^{+}$ | 470 |
| $K^{+}$ | 10.2 |
| $Ca^{2+}$ | 10.3 |
| $Mg^{2+}$ | 53 |
| $Sr^{+}$ | 0.09 |
| $Cl^{-}$ | 521 |
| $SO_4^{2-}$ | 23 |
| Trace elemental | Concentration (μmol/Kg) |
| Li | 54 |
| Si | 16 |
| Mo | 1.8 |
| Ba | 0.85 |
| V | 2.9 |
| Ni | 1.7 |
| Cr | 7.5 |
| Al | 240 |
| Cu | 1.8 |
| Zn | 0.5 |
| Mn | 1.2 |
| Fe | 0.24 |
| Cd | 0.24 |
| Pb | 2.1 |
| Co | 1.3 |
| Ag | 2.3 |
| Ti | 0.67 |

**Table S2** Kinetic rate laws used in COMSOL

| Rate Constant | Value | Unit | Ref. |
|---|---|---|---|
| $k_{f1}$ | $3.71 \times 10^{-2}$ | $s^{-1}$ | 3 |
| $k_{f2}$ | $2.23 \times 10^{3}$ | $M^{-1}s^{-1}$ | 3 |
| $k_{f3}$ | $5 \times 10^{10}$ | $M^{-1}s^{-1}$ | 4 |
| $k_{f4}$ | $9 \times 10^{4}$ | $s^{-1}$ | $k_{r2} \times k_{f1} \times k_{f3}$ |
| $k_{r1}$ | $7.83 \times 10^{4}$ | $M^{-1}s^{-1}$ | 3 |
| $k_{r2}$ | $4.85 \times 10^{-5}$ | $s^{-1}$ | 3 |
| $k_{r3}$ | $2.5$ | $s^{-1}$ | 3 |
| $k_{r4}$ | $4.37 \times 10^{5}$ | $s^{-1}$ | $k_{f2} \times k_{r1} \times k_{r3}$ |
| $k_{fw}$ | $1.4 \times 10^{-3}$ | $M\,s^{-1}$ | 4 |
| $k_{rw}$ | $1.6 \times 10^{11}$ | $M^{-1}s^{-1}$ | $k_{fw}/K_w$ |

To validate the reliability of kinetic rates used in this work. We simulated the fraction of dissolved inorganic carbon species i.e., carbonate, bicarbonate, and carbon dioxide at different pHs under equilibrium by using the kinetic rates in Table S2. The simulation based on kinetics agrees with the thermodynamics-based Bjerrum plot (Fig. S40), suggesting that our approach is very applicable to the non-equilibrium photoelectrochemical processes.

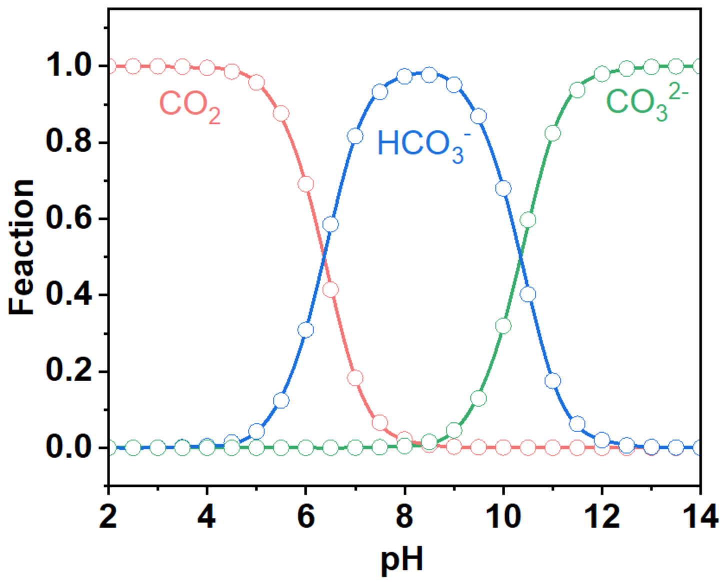


**Fig. S40** Comparison of simulation results (open circles) with Bjerrum plot (solid lines).

**Table S3** Diffusion coefficient used in COMSOL

| Diffusion Coefficient | Value | Unit |
|---|---|---|
| $\boldsymbol{D_{H^+}}$ | $9.31 \times 10^{-9}$ | $m^2 s^{-1}$ |
| $\boldsymbol{D_{OH^-}}$ | $5.27 \times 10^{-9}$ | $m^2 s^{-1}$ |
| $\boldsymbol{D_{CO_2}}$ | $1.9 \times 10^{-9}$ | $m^2 s^{-1}$ |
| $\boldsymbol{D_{HCO_3^-}}$ | $1.18 \times 10^{-9}$ | $m^2 s^{-1}$ |
| $\boldsymbol{D_{CO_3^{2-}}}$ | $0.955 \times 10^{-9}$ | $m^2 s^{-1}$ |

**Table S4** Electric charge of species used in COMSOL

| Charge of Species | Value |
|---|---|
| $\boldsymbol{Z_{H^+}}$ | 1 |
| $\boldsymbol{Z_{OH^-}}$ | -1 |
| $\boldsymbol{Z_{CO_2}}$ | 1 |
| $\boldsymbol{Z_{HCO_3^-}}$ | -1 |
| $\boldsymbol{Z_{CO_3^{2-}}}$ | -2 |
| $\boldsymbol{Z_{H_2O}}$ | 0 |

**Table S5** PEC performance of recently reported silicon photocathodes for $CO_2$RR

| Photocathode | Onset potential (V vs RHE) | Electrolyte / pH | FE (%) | Irradiation Intensity | Ref |
|---|---|---|---|---|---|
| **$p^+$-a-Si/Si/ $n^+$-a-Si/Ag/Au** | **0.75** | **Sea water** | **73 for CO** | **1 Sun** | **This work** |
| Si/$TiO_2$/ APTES* | 0 | $CO_2$ saturated 0.1 M $KHCO_3$ | 76 for CO | 1.5 Suns | 5 |
| Si/Au | 0.22 | $CO_2$ saturated 0.1 M $KHCO_3$ | 82.2 for CO | 1 Sun | 6 |
| $n^+$p-Si/Au/$TiO_2$ | 0.24 | $CO_2$ saturated 0.1 M $KHCO_3$ | 86 for CO | 1 Sun | 7 |
| Si/Cu | 0 | $CO_2$ saturated 0.1 M $KHCO_3$ | 25 for $C_2H_4$ | 1 Sun | 8 |
| Si/$TiO_2$/CNT/@$Co^{II}$ -(BrqPy)* | 0.2 | $CO_2$ saturated 0.1 M $KHCO_3$ | 100 for CO | 1 Sun | 9 |
| p-Si/n-$ZnO_v$/p-$Cu_xO$ | 0.2 | $CO_2$ saturated 0.1 M $KHCO_3$ | 60 for $C_2H_5OH$ | 1 Sun | 10 |
| p-Si/ZnO/Bi-$Bi_2O_3$ | -0.3 | $CO_2$ saturated 0.1 M $KHCO_3$ | 84.3 for formate | 2 Suns | 11 |
| p-Si/Ni@In | -0.5 | $CO_2$ saturated 0.1 M $KHCO_3$ | 87 for formate | 1 Sun | 12 |
| p-Si/$SiO_2$-Cu-Ag | -0.8 | $CO_2$ saturated 0.1 M $KHCO_3$ | 65.9 for CO | 1 Sun | 13 |
| $n^+$p-Si/Cu | 0.2 | $CO_2$ saturated 0.1 M $KHCO_3$ | Not reported | 1 Sun | 14 |
| b-Si/Ag/2ABT* | 0 | $CO_2$ saturated 0.2 M $KHCO_3$ | 75 for CO | 0.66 Sun | 15 |
| p-Si/Bi | 0.1 | $CO_2$ saturated 0.5 M $KHCO_3$ | 72 for formate | 1 Sun | 16 |
| p-Si/$TiO_2$/Ag | -0.16 | $CO_2$ saturated 0.1 M $KHCO_3$ | 47 for CO | 1 Sun | 17 |
| p-Si/Bi | -0.2 | $CO_2$ saturated 0.5 M $KHCO_3$ | 90 for formate | 0.5 Sun | 18 |
| $n^+pp^+$-Si/Ag-Cu | 0.1 | $CO_2$ saturated 0.1 M CsHCO3 | 60 for hydrocarbon | 1 Sun | 19 |
| p-Si/Bi | 0 | $CO_2$ saturated 0.5 M $KHCO_3$ | 90 for formate | 0.5 Sun | 20 |
| p-Si/Ag | -0.6 | $CO_2$ saturated 0.1 M $KHCO_3$ | 53 for CO | 1 Sun | 21 |
| $n^+$p-Si/Sn-pNWs* | 0.32 | $CO_2$ saturated 0.1 M $KHCO_3$ | 59.2 for HCOOH | 1 Sun | 22 |
| p-Si/Ag | 0 | $CO_2$ saturated 0.5 M $KHCO_3$ | 90 for CO | 0.5 Sun | 23 |
| p-Si/N-GQSs | -1.53 vs Ag/$Ag^+$ | $CO_2$ saturated 0.1 M TBAH | 95 for CO | 1 Sun | 24 |

*APTES: (3-aminopropyl)triethoxysilane
*(BrqPy): 4’,4”-bis(4-bromophenyl)-2,2’ :6’,2” :6”,2’”-quaterpyridine)
*DCP: 2,6-dicarboxypyridin-4-yl
*ABT: 2-aminobenzenethiol
*Sn-pNWs: porous $SnO_2$ nanowires
*N-GQSs: nitrogen-doped graphene quantum sheets

**Table S6** Performance of recently reported unbiased cells ($BiVO_4$) for $CO_2RR$

| Photoanodes | Photocathodes | Catalysts | Solution | pH | STF (%) | Ref. |
|---|---|---|---|---|---|---|
| $BiVO_4$ | Perovskite | CoMTPP* | $CO_2$ saturated 0.5 M $KHCO_3$ | 7.4 | 0.63 | [25] |
| $BiVO_4$ | Perovskite | CotpyP* | $CO_2$ saturated 0.1 M $KHCO_3$ | 6.7 | 0.08 | [26] |
| $BiVO_4$ | Perovskite | S.ovata | S. ovatamedium | 7 | 0.7 | [27] |
| $BiVO_4$ | a-Si | Au | $CO_2$ saturated 0.1 M $KHCO_3$ | 6.8 | 0.43 | [28] |
| $BiVO_4$ | CuO | ClFDH* | $CO_2$ saturated 0.1 M phosphate buffer | 6.5 | 0.008 | [29] |
| **$BiVO_4$** | **Si** | **Au** | **Sea water without $CO_2$ saturated** | **8.3** | **0.71** | **This work** |

*CoMTPP: cobalt(II) meso-tetrakis(4-methoxyphenyl)-porphyrin

*CotpyP: phosphonated cobalt(II) bis(terpyridine)

*ClFDH: Clostridium ljungdahlii

**References:**


1 Wu, B. *et al.* Stable solar water splitting with wettable organic-layer-protected silicon photocathodes. *Nature Communications* **13**, doi:ARTN 446010.1038/s41467-022-32099-1 (2022).

2 Bae, D., Seger, B., Vesborg, P. C., Hansen, O. & Chorkendorff, I. Strategies for stable water splitting via protected photoelectrodes. *Chem Soc Rev* **46**, 1933-1954, doi:10.1039/c6cs00918b (2017).

3 Chen, Y., Lewis, N. S. & Xiang, C. Modeling and Simulation of the Spatial and Light-Intensity Dependence of Product Distributions in an Integrated Photoelectrochemical CO2 Reduction System. *ACS Energy Letters* **1**, 273-280, doi:10.1021/acsenergylett.6b00134 (2016).

4 Schulz, K. G., Riebesell, U., Rost, B., Thoms, S. & Zeebe, R. E. Determination of the rate constants for the carbon dioxide to bicarbonate inter-conversion in pH-buffered seawater systems. *Marine Chemistry* **100**, 53-65, doi:10.1016/j.marchem.2005.11.001 (2006).

5 Shang, B. *et al.* Aqueous Photoelectrochemical CO(2) Reduction to CO and Methanol over a Silicon Photocathode Functionalized with a Cobalt Phthalocyanine Molecular Catalyst. *Angew Chem Int Ed Engl* **62**, e202215213, doi:10.1002/anie.202215213 (2023).

6 Hu, J. *et al.* Facet engineering in Au nanoparticles buried in p-Si photocathodes for enhanced photoelectrochemical CO2 reduction. *Applied Catalysis B: Environmental* **327**, doi:10.1016/j.apcatb.2023.122438 (2023).

7 Wang, K. *et al.* Steering the Pathway of Plasmon-Enhanced Photoelectrochemical CO(2) Reduction by Bridging Si and Au Nanoparticles through a TiO(2) Interlayer. *Small* **18**, e2201882, doi:10.1002/smll.202201882 (2022).

8 Roh, I. *et al.* Photoelectrochemical CO(2) Reduction toward Multicarbon Products with Silicon Nanowire Photocathodes Interfaced with Copper Nanoparticles. *J Am Chem Soc* **144**, 8002-8006, doi:10.1021/jacs.2c03702 (2022).

9 Wen, Z. *et al.* Aqueous CO(2) Reduction on Si Photocathodes Functionalized by Cobalt Molecular Catalysts/Carbon Nanotubes. *Angew Chem Int Ed Engl* **61**, e202201086, doi:10.1002/anie.202201086 (2022).

10 Kan, M. *et al.* Defect-Assisted Electron Tunneling for Photoelectrochemical CO2 Reduction to Ethanol at Low Overpotentials. *Advanced Energy Materials* **12**, doi:10.1002/aenm.202201134 (2022).

11 Zhang, Q. *et al.* A Bismuth Species-Decorated ZnO/p-Si Photocathode for High Selectivity of Formate in CO2 Photoelectrochemical Reduction. *ACS Sustainable Chemistry & Engineering* **10**, 2380-2387, doi:10.1021/acssuschemeng.1c06712 (2022).

12 Ma, W. *et al.* Nickel and indium core-shell co-catalysts loaded silicon nanowire arrays for efficient photoelectrocatalytic reduction of CO2 to formate. *Journal of Energy Chemistry* **54**, 422-428, doi:10.1016/j.jechem.2020.06.023 (2021).

13 Dong, W. J. *et al.* Grain Boundary Engineering of Cu-Ag Thin-Film Catalysts for Selective (Photo)Electrochemical CO(2) Reduction to CO and CH(4). *ACS Appl Mater Interfaces* **13**, 18905-18913, doi:10.1021/acsami.1c03735 (2021).

14 Kempler, P. A., Richter, M. H., Cheng, W.-H., Brunschwig, B. S. & Lewis, N. S. Si

Microwire-Array Photocathodes Decorated with Cu Allow CO2 Reduction with Minimal Parasitic Absorption of Sunlight. *ACS Energy Letters* **5**, 2528-2534, doi:10.1021/acsenergylett.0c01334 (2020).

15 Kan, M. *et al.* 2-Aminobenzenethiol-Functionalized Silver-Decorated Nanoporous Silicon Photoelectrodes for Selective CO(2) Reduction. *Angew Chem Int Ed Engl* **59**, 11462-11469, doi:10.1002/anie.202001953 (2020).

16 Fu, D. *et al.* Bismuth-Decorated Silicon Photocathodes for CO2-to-Formate Solar-Driven Conversion. *ChemCatChem* **12**, 5819-5825, doi:10.1002/cctc.202000889 (2020).

17 Kim, C. *et al.* Photoelectrochemical Reduction of CO2 to Syngas by Reduced Ag Catalysts on Si Photocathodes. *Applied Sciences* **10**, doi:10.3390/app10103487 (2020).

18 Ding, P. *et al.* Controlled chemical etching leads to efficient silicon–bismuth interface for photoelectrochemical CO2 reduction to formate. *Materials Today Chemistry* **11**, 80-85, doi:10.1016/j.mtchem.2018.10.009 (2019).

19 Gurudayal, G. *et al.* Si photocathode with Ag-supported dendritic Cu catalyst for CO2reduction. *Energy & Environmental Science* **12**, 1068-1077, doi:10.1039/c8ee03547d (2019).

20 Gong, Q. *et al.* Structural defects on converted bismuth oxide nanotubes enable highly active electrocatalysis of carbon dioxide reduction. *Nat Commun* **10**, 2807, doi:10.1038/s41467-019-10819-4 (2019).

21 Wei, L. *et al.* Photoelectrocatalytic reduction of CO2 to syngas over Ag nanoparticle modified p-Si nanowire arrays. *Nanoscale* **11**, 12530-12536, doi:10.1039/c9nr02786f (2019).

22 Rao, K. R. *et al.* Photoelectrochemical reduction of CO2to HCOOH on silicon photocathodes with reduced SnO2porous nanowire catalysts. *Journal of Materials Chemistry A* **6**, 1736-1742, doi:10.1039/c7ta09672k (2018).

23 Hu, Y. *et al.* Designing effective Si/Ag interface via controlled chemical etching for photoelectrochemical CO2 reduction. *Journal of Materials Chemistry A* **6**, 21906-21912, doi:10.1039/c8ta05420g (2018).

24 Yang, K. D. *et al.* Graphene Quantum Sheet Catalyzed Silicon Photocathode for Selective CO2 Conversion to CO. *Advanced Functional Materials* **26**, 233-242, doi:10.1002/adfm.201502751 (2015).

25 Andrei, V. *et al.* Floating perovskite-BiVO(4) devices for scalable solar fuel production. *Nature* **608**, 518-522, doi:10.1038/s41586-022-04978-6 (2022).

26 Wang, Q. *et al.* Molecularly engineered photocatalyst sheet for scalable solar formate production from carbon dioxide and water. *Nature Energy* **5**, 703-710, doi:10.1038/s41560-020-0678-6 (2020).

27 Wang, Q., Kalathil, S., Pornrungroj, C., Sahm, C. D. & Reisner, E. Bacteria-photocatalyst sheet for sustainable carbon dioxide utilization. *Nature Catalysis* **5**, 633-+, doi:10.1038/s41929-022-00817-z (2022).

28 Li, C. *et al.* Photoelectrochemical CO2 reduction to adjustable syngas on grain-boundary-mediated a-Si/TiO2/Au photocathodes with low onset potentials. *Energy & Environmental Science* **12**, 923-928, doi:10.1039/c8ee02768d (2019).

29 Kuk, S. K. *et al.* CO(2) -Reductive, Copper Oxide-Based Photobiocathode for Z-

Scheme Semi-Artificial Leaf Structure. *ChemSusChem* **13**, 2940-2944, doi:10.1002/cssc.202000459 (2020).
