# Supplementary material for: Solar-driven selective conversion of millimolar dissolved carbon to fuels with molecular flux generation
Source: Nat Commun. 2025 Feb 12;16:1558. doi: 10.1038/s41467-025-56106-3 (PMC11821833; doi:10.1038/s41467-025-56106-3)
Supplement: Supplementary file 2 — Description of Additional Supplementary Files [file 41467_2025_56106_MOESM2_ESM.pdf]

Description of Additional Supplementary Information

Supplementary Movie 1: Visualized flow field test using black ink

Supplementary Movie:2: PEC CO_2 capture and conversion in sea water
